# Possible involvement of silent mutations in cancer pathogenesis and evolution

**DOI:** 10.1038/s41598-023-34452-w

**Published:** 2023-05-10

**Authors:** Chie Kikutake, Mikita Suyama

**Affiliations:** Division of Bioinformatics, Medical Institute of Bioregulation, Kyushu University, Fukuoka, 812-8582 Japan

**Keywords:** Cancer, Computational biology and bioinformatics, Genetics

## Abstract

Recent studies have shown that some silent mutations can be harmful to various processes. In this study, we performed a comprehensive in silico analysis to elucidate the effects of silent mutations on cancer pathogenesis using exome sequencing data derived from the Cancer Genome Atlas. We focused on the codon optimality scores of silent mutations, which were defined as the difference between the optimality of synonymous codons, calculated using the codon usage table. The relationship between cancer evolution and silent mutations showed that the codon optimality score of the mutations that occurred later in carcinogenesis was significantly higher than of those that occurred earlier. In addition, mutations with higher scores were enriched in genes involved in the cell cycle and cell division, while those with lower scores were enriched in genes involved in apoptosis and cellular senescence. Our results demonstrate that some silent mutations can be involved in cancer pathogenesis.

## Introduction

A silent mutation alters the nucleotide sequence but not the amino acid sequence. Silent mutations have been considered neutral or nearly neutral because, unlike missense mutations or indels, they do not affect protein function^1^. Recently, however, several studies have shown that silent mutations may potentially be harmful^2–12^. Silent mutations can affect on protein levels or conformation by modifying mRNA stability, miRNA binding sites, translation efficiency or splicing regulatory sites^13,14^. We used the term "mutation" as somatic mutation in this study.

Silent mutations can affect codon optimality: the non-uniform abundance of the 61 amino acid-encoding codons translated by ribosomes^2^. Each codon encoding the same amino acid has a different codon optimality. Optimal codons correspond to more abundant tRNA in cells and more abundant codons in transcripts. They are associated with higher mRNA stability, faster translation speed, higher translation efficiency, and higher fidelity of protein folding^3–5^. Introducing non-optimal codons into yeast genes has been shown to slow down translation and significantly decrease mRNA stability, suggesting a link between translation dynamics and mRNA decay^3,6,7^. Another study constructed approximately 8000 yeast mutants carrying a synonymous, nonsynonymous, or nonsense mutation and measured their fitness relative to the wild-type^8^. Three-quarters of the synonymous mutations caused a significant reduction in fitness, and the distribution of fitness effects was similar for both synonymous and nonsynonymous mutations^8^. Also, both these types of mutations frequently disturbed mRNA expression of the mutated gene^8^.

The change in codon optimality induced by silent mutations can be important in cancer cell growth^9–12^. Silent mutations giving rise to optimal codons were found enriched in oncogenes, leading to greater translational efficiency in liver cancer tissues compared with normal tissues. Similarly, silent mutations resulting in non-optimal codons were found enriched in tumor suppressor genes, leading to lower translational efficiency^9–11^. Silent mutations in *BRCA1*, a tumor suppressor, were found to decrease its codon optimality, consequently attenuating its activity in HAP1 cells^12^. Despite these studies, the relationship between changes in codon optimality and cancer pathogenesis remains poorly understood. Missense mutations are actively studied in cancer cells, while the effect of silent mutations is usually underestimated or overlooked.

The aim of this study was to elucidate the impact of silent mutations on cancer pathogenesis. We performed a comprehensive in silico analysis of silent mutations using somatic mutation data on 33 cancer types derived from the Cancer Genome Atlas (TCGA). We examined the changes in codon optimality induced by these mutations and constructed a silent mutation model in cancer evolution. Our results indicate that silent mutations are not necessarily neutral, but are likely to occur throughout the evolution of cancer to support its drastic growth and progression.

## Results

### Characteristics of silent mutations

**Figure 1:**
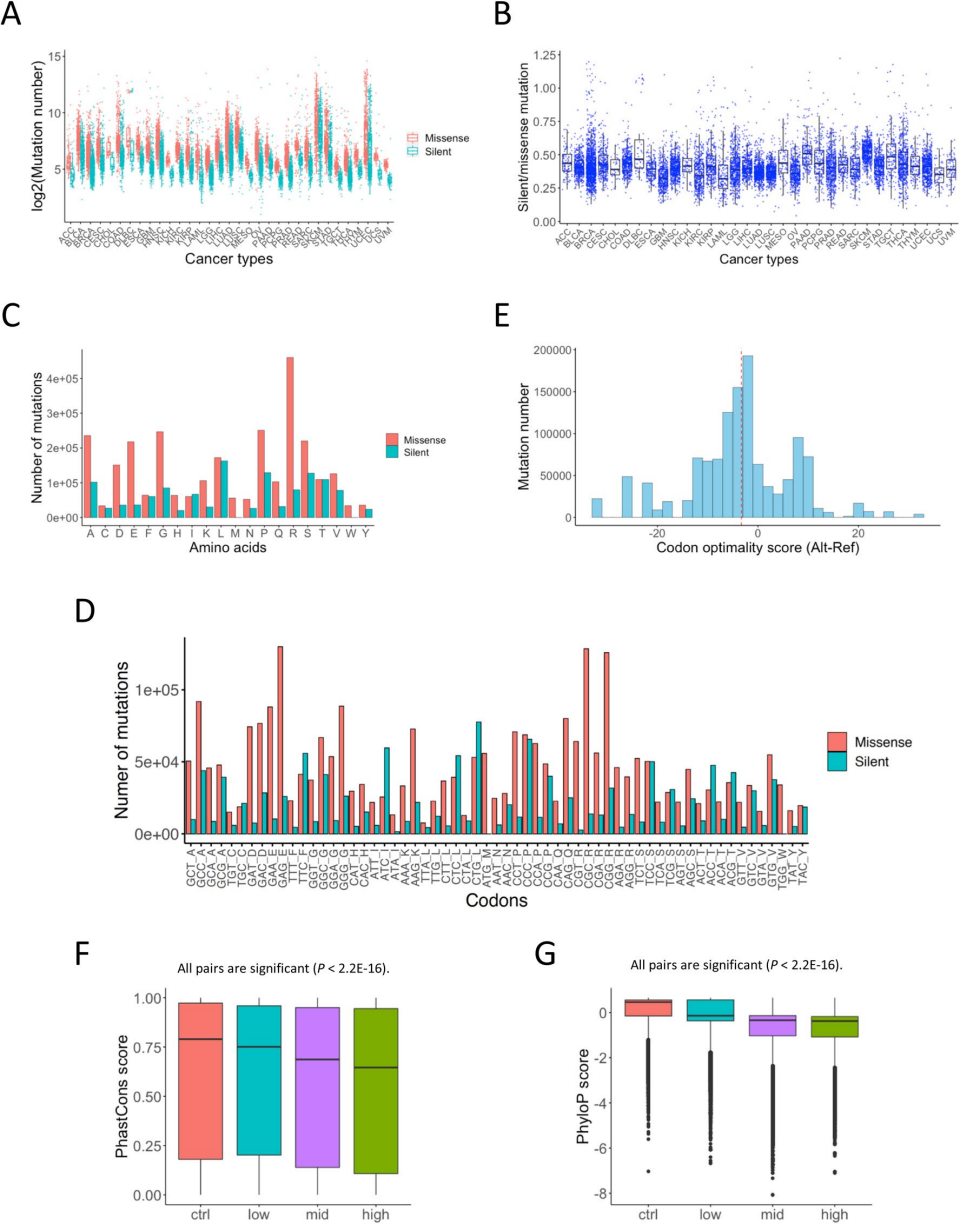
Characteristics of silent mutations and their codon optimality score. (**A**) The total number of missense mutations and silent mutations in each sample of each cancer type. (**B**) The ratio of the total number of silent mutations to missense mutations in each sample of each cancer type. (**C**) The number of silent mutations per amino acid. The horizontal axis represents the 20 amino acids. (**D**) The number of silent mutations per codon. The horizontal axis represents the 61 amino acid-encoding codons. (**E**) The distribution of codon optimality scores for each silent mutation. The horizontal axis represents the codon optimality score calculated as the frequency of the codon with mutation (Alt) minus the frequency of the corresponding codon without mutation (Ref). The vertical axis represents the number of silent mutations. The red dotted lines represent the median of the codon optimality score. (**F**) The distribution of PhastCons scores for each silent mutation. The horizontal axis represents a control group and three groups based on the codon optimality scores of silent mutations (low: < − 10, mid: ≥ − 10 and < 10, and high: ≥ 10). The control group represents 100,000 bases randomly selected from the coding sequence. The Wilcoxon rank sum test for every pair showed a significant difference (*P* < 2.2e−16). (**G**) The distribution of PhyloP scores for each silent mutation. The horizontal axis represents a control group and three groups based on the codon optimality scores of silent mutations (low: < − 10, mid: ≥ − 10 and < 10, and high: ≥ 10). The control group represents 100,000 bases randomly selected from the coding sequence. The Wilcoxon rank sum test for every pair showed a significant difference (*P* < 2.2e−16).

To analyze silent mutations in coding regions from cancer samples, we used the whole exome sequencing (WES) data from TCGA, which were obtained from 10,437 samples of 33 cancer types (Supplementary Table 1). These mutation data are publicly available, variant-called, and accumulated by the Multi-Center Mutation Calling in Multiple Cancers (MC3) Project^15^. The total numbers of silent and missense mutations were 1,238,725 and 2,888,649, respectively (Supplementary Table 1). The median ratios of the number of silent mutations to missense mutations were similar for all cancer types (0.320–0.523) (Supplementary Table 1, Fig. 1A,B).

To evaluate bias in the codons with mutations, we first examined the frequency of each amino acid with mutations. We found that missense mutations occurred more frequently in R (arginine)-encoding codons, while silent mutations were found more frequently in L (leucine)-encoding codons (Fig. 1C). Both these residues are encoded by six different codons each. Then, we examined the number of mutations in each codon. The missense mutations occurred more frequently in the GAG, CGC, and CGG codons (Fig. 1D), corresponding to well-known driver mutations in *PIK3CA* (E545) and *TP53* (R175 and R248)^9^. The silent mutations occurred more frequently in the CTG, CCC, and ATC codons. Both these types of mutations are likely to occur in a particular position, and some of them may have an impact on cancer pathogenesis.

Next, we determined the codon optimality score (alt—ref) of codons with silent mutations (Fig. 1E); those with positive scores were defined as “optimal mutations” and those with negative scores as “non-optimal mutations.” The median codon optimality score was − 3.30 for all the silent mutations, implying that 68.8% of their scores were negative (Fig. 1E). Using silent single nucleotide polymorphisms (SNPs) registered in gnomAD, we found that the codon optimality scores of silent SNPs with high allele frequency (≥ 0.01) were skewed slightly more toward positive than those of silent SNPs with low allele frequency (< 0.01) (Supplementary Fig. S1A,B). These results suggest that optimal mutations may be advantageous to the population and more likely to be fixed in the population. To further confirm the association between the evolutionary conservation of bases harboring silent mutations in cancer and their codon optimality scores, we divided silent mutations into three groups based on their scores (low: < − 10, mid: ≥ − 10 and < 10, and high: ≥ 10). Comparing PhastCons and PhyloP scores of silent mutations among the three groups plus the control group (100,000 bases randomly selected from the coding sequence [CDS]), the codon optimality scores of silent mutations were negatively correlated with their evolutionary conservation (Fig. 1F,G). These results indicate that the originally high-frequency codons are functionally and evolutionarily more important than the originally low-frequency codons.

### Timing of silent mutations in cancer evolution

**Figure 2:**
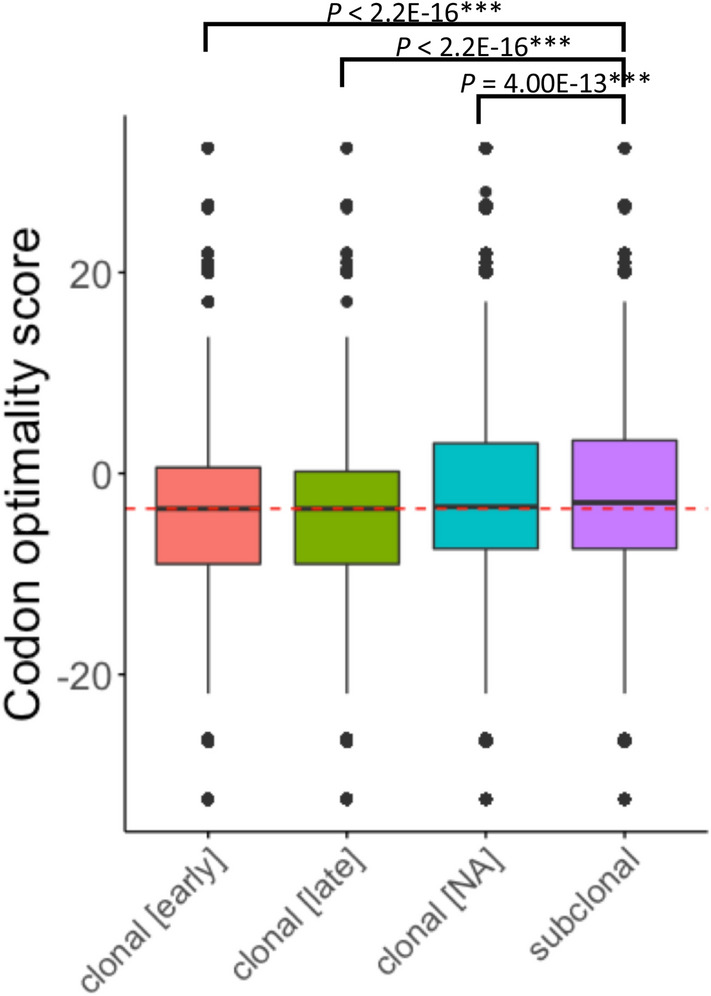
Codon optimality score of silent mutations in cancer evolution. The evolutionary group for silent mutations and their codon optimality scores. The horizontal axis represents the four evolutionary groups (clonal [early] = Early clonal, clonal [late] = Late clonal, clonal [NA] = Clonal, and subclonal = Subclonal) classified by MutationTimeR.

Several clones with different mutations are generated through the evolutionary process of cancer, resulting in intratumor heterogeneity^16^. Different evolutionary stages of cancer are associated with different mutation patterns^17^. Therefore, to examine whether the codon optimality scores of silent mutations also change with the evolutionary process of cancer, we examined the clonal allelic status of each silent mutation using MutationTimeR (https://github.com/gerstung-lab/MutationTimeR), a method to estimate the timing of somatic mutations relative to clonal and subclonal copy number states and to calculate the relative timing of copy number gains^17^. We found that the codon optimality scores significantly increased in the subclonal group compared with the early clonal, late clonal, and clonal groups (Fig. 2). These results suggest that optimal mutations are prone to occur at the later stages in cancer evolution, leading to promote translation processes, such as mRNA stability, translation efficiency, and protein folding.

### Silent mutations and their surrounding nucleotide context

**Figure 3:**
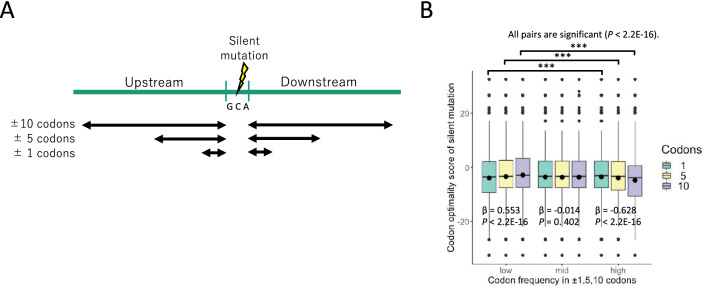
Surrounding nucleotide context of silent mutations. (**A**) Schematic diagram of the context of nucleotides surrounding the codons with silent mutations. The nucleotide context was obtained for three different codon lengths upstream and downstream of the silent mutation-carrying codons. (**B**) The mean codon frequencies (per 1,000 triplets) of ± 1, ± 5, and ± 10 codons from the silent mutation-carrying codons and their codon optimality scores. The horizontal axis represents three groups based on the mean codon frequencies of the ± 1, ± 5, and ± 10 codons (low: bottom 25%, mid: 25–75%, and high: top 25%).

Based on the assumption that silent mutations can affect translation efficiency, we hypothesized that they share a relationship with their surrounding nucleotides. To confirm the characteristics of codons upstream and downstream of silent mutations, we analyzed the sequences of ± 3 bases (± 1 codons), ± 15 bases (± 5 codons), and ± 30 bases (± 10 codons) using the human protein-coding sequence data in the Consensus CDS (CCDS) project (Fig. 3A). Codons with silent mutations were classified into three groups based on their average codon frequencies: the top 25% (high), the bottom 25% (low), and the rest (mid). Among the ± 10 codons, the high group had significantly lower codon optimality scores than the low group (median, mean: − 3.80, − 4.73 vs. − 2.90, − 2.83) (Fig. 3B). Although similar results were obtained upon analyzing the ± 5 codons (3.30, − 3.90 vs. − 3.30, − 3.43), the opposite trend was observed in the ± 1 codons (− 3.10, − 3.51 vs. − 3.50, − 3.92). The codon optimality scores of silent mutations in the low group were positively correlated with the average frequencies (per 1,000 triplets) of the ± 1, ± 5, and ± 10 codons, while those of silent mutations in the high group were negatively correlated with the average frequencies of the neighboring codons. Similar results were obtained when the upstream and downstream codons with silent mutations were analyzed separately (Supplementary Fig. S2A,B). These results suggest that in regions rich in low-frequency codons, silent mutations with higher codon optimality scores may increase the efficiency of translation processes, whereas in regions rich in high-frequency codons, silent mutations with lower codon optimality scores may decrease the efficiency of translation.

### Silent mutations possibly associated with cancer pathogenesis

In addition to mutation data, RNA-Seq data from the corresponding samples are available via TCGA data repository. Using this RNA-Seq data, we explored the silent mutations that can cause codon-mediated mRNA decay^18^ due to ribosome slowdown. First, to examine the silent mutations with potential impact on exonic splicing elements, we extracted silent mutations located in splicing elements^19^. We found only 10 silent mutations had the potential to affect splicing. Therefore, the following analysis was performed without removing mutations that might affect splicing mechanisms. Then, we focused on recurrent silent mutations that were found in ≥ 3 samples without distinguishing them by cancer type based on the rationale that they may influence cancer development, considering that the likelihood of mutations occurring by chance at the same position in the CDS is statistically low^20^. Also, we wanted to ensure a sufficient sample size for the following differential gene expression analysis. We extracted 45,003 recurrent silent mutations in 13,580 genes and divided them into three groups based on the codon optimality scores (low: < − 10, mid: ≥ − 10 and < 10, and high: ≥ 10). Silent mutations in the high or low groups were shared by significantly more samples than those in the mid group (Supplementary Fig. S3A). Some of those, with extremely high or low codon optimality scores, may have an important role in cancer cells.

To identify silent mutations associated with cancer pathogenesis, we selected those with absolute codon optimality scores ≥ 10, yielding 8229 candidate mutations in 5411 genes. Among them, we examined the expression levels of the corresponding genes in every cancer type and further extracted those that satisfied the following criteria: genes significantly differentially expressed between samples with mutations and samples without mutations (e.g. Concerning gene A, we compared expression levels of gene A between samples with mutations in gene A and samples without mutations in gene A), genes with mean expression levels of log_10_(transcripts per kilobase million [TPM]) ≥ 1, and genes with recurrent mutations found in ≥ 3 samples by distinguishing cancer type. Finally, we obtained 16 mutations with a false discovery rate < 0.25 (Supplementary Table 2)^21^. Although we found that only *MAML2* and *YY1AP1* were included in the oncogene list, no missense or loss-of-function mutations were observed in all samples with the silent mutations in *MAML2* or *YY1AP1*. Moreover, we investigated the co-occurrence of other types of mutations that could influence RNA expression levels. Although missense mutations were observed in all samples with the silent mutations in *FANCA* and *PDIA2*, they were not recurrent mutations. Of the 16 genes, eight were non-optimal mutations that had significantly reduced the expression of the corresponding genes, and two were optimal mutations that had significantly increased the expression of the corresponding genes. The silent mutations in the 16 genes are recurrent, which is a very low probability when we assume those mutations occur randomly. Therefore, we believe that silent mutations in these genes may have some effect on cancer cells.

**Figure 4:**
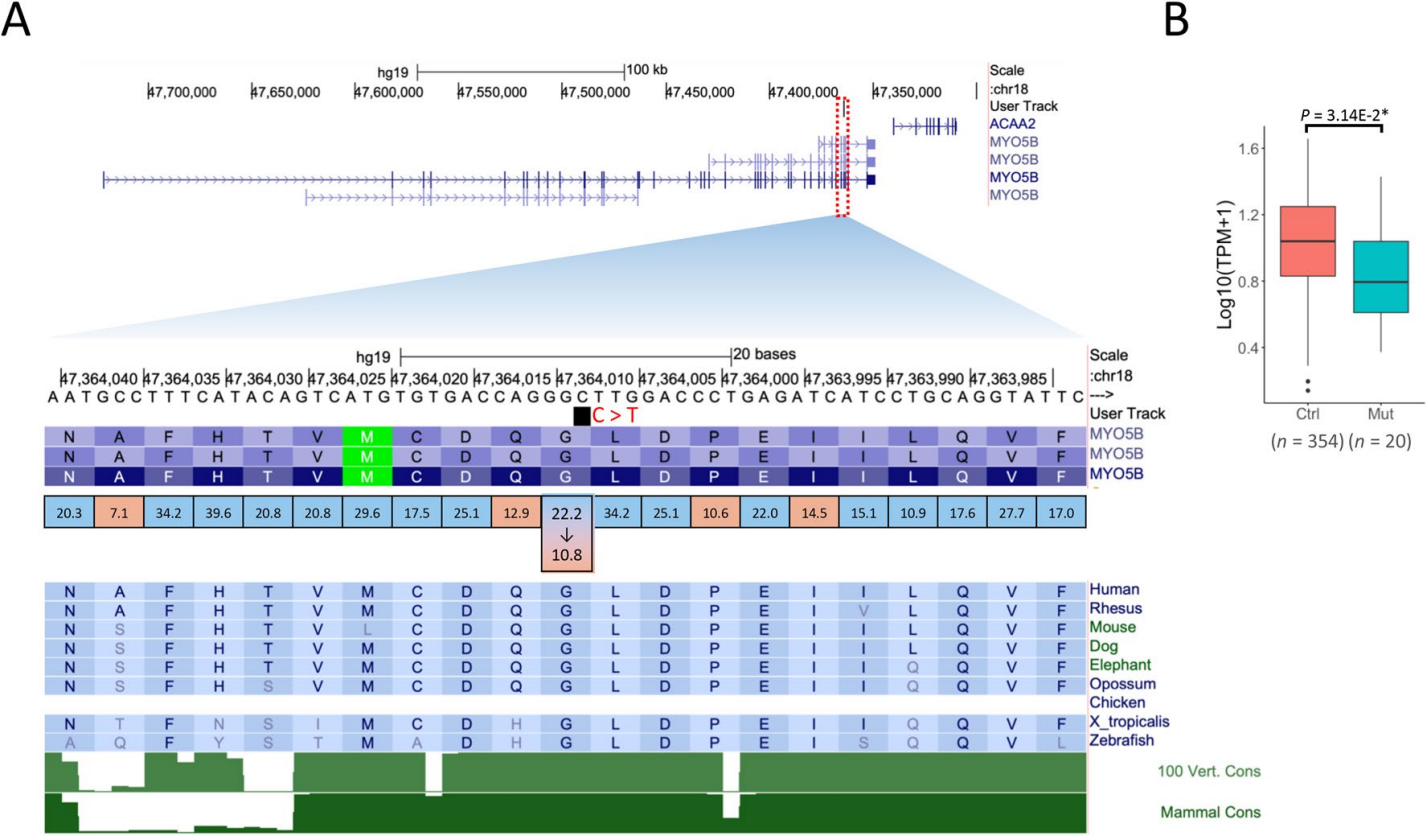
Association between silent mutation in *MYO5B* and cancer pathogenesis. (**A**) The position of the extracted silent mutation in *MYO5B*. The figure shows nucleotide sequence, amino acid sequence, codon optimality, and evolutionary conservation status. Numbers in squares are codon frequencies (per 1000 triplets). The blue squares indicate codons ≥ 15.1 (median frequency) and red squares indicate codons < 15.1. (**B**) The expression levels of *MYO5B* with and without the silent mutation.

One of the mutations was in *MYO5B* (myosin VB) found in OV (*n* = 20) (Fig. 4A). High-frequency codons were relatively abundant in both upstream and downstream sequences. The silent mutation GGC > GGT reduced the codon optimality score from 22.2 to 10.8. This variant is not registered in the ClinVar database^22^. The nucleotide was evolutionarily conserved among mammals. The expression of *MYO5B* was significantly lower in the group with the mutation than in those without it (*P* = 0.0314) (Fig. 4B). MYO5B belongs to the large myosin family of actin-based molecular motor proteins, known to control multiple trafficking pathways^23^. It has been implicated in the establishment of polarized function in epithelial cells^24^. Indeed, loss-of-function *MYO5B* mutations cause neonatal diarrheal disease in children with microvillus inclusion disease, likely due to defects in apical polarization and transporter protein localization^25,26^. Its inactivation promotes the proliferation, invasion, and migration of gastric cancer cells^27^. Although the function of *MYO5B* in OV remains unclear, the extracted silent mutation may possibly decrease its protein levels, promoting the growth of OV, as in gastric cancer.

Another silent mutation, CAG > CAA, was identified in *RAI1* (retinoic acid induced 1) found in PCPG (*n* = 4) (Supplementary Fig. 3B). Again, high-frequency codons were accumulated upstream and downstream of the mutation and it reduced the codon optimality score from 34.2 to 12.3. In the ClinVar database^22^, this variant is annotated as “benign/likely benign.” It is possible that this annotation is due to the silent/synonymous mutation. Therefore, it is unclear whether this silent mutation could have an effect on the disease. *RAI1* was expressed at a significantly lower level in samples with the mutation compared with those without it (*P* = 0.0103) (Supplementary Fig. 3C). The decreased expression of both these genes may be due to codon-mediated mRNA decay caused by silent mutation-induced ribosome slowdown^18^. RAI1 is a nuclear protein whose structure and sequence are conserved across model vertebrates^28,29^. Haploinsufficiency of RAI1 causes Smith–Magenis syndrome, which is associated with diverse neurodevelopmental and behavioral symptoms^30^. *RAI1* is expressed in various cell lines, tissues, and tumor cells, and functions as a tumor suppressor in esophageal cancer with a strong ability to predict patient survival^31^. Like MYO5B, the silent mutation in *RAI1* could decrease its protein levels, leading to the progression of PCPG. These results suggest that some silent mutations can be pathogenic and may have harmful effects on cancer cells.

### Function and expression of genes with silent mutations

**Figure 5:**
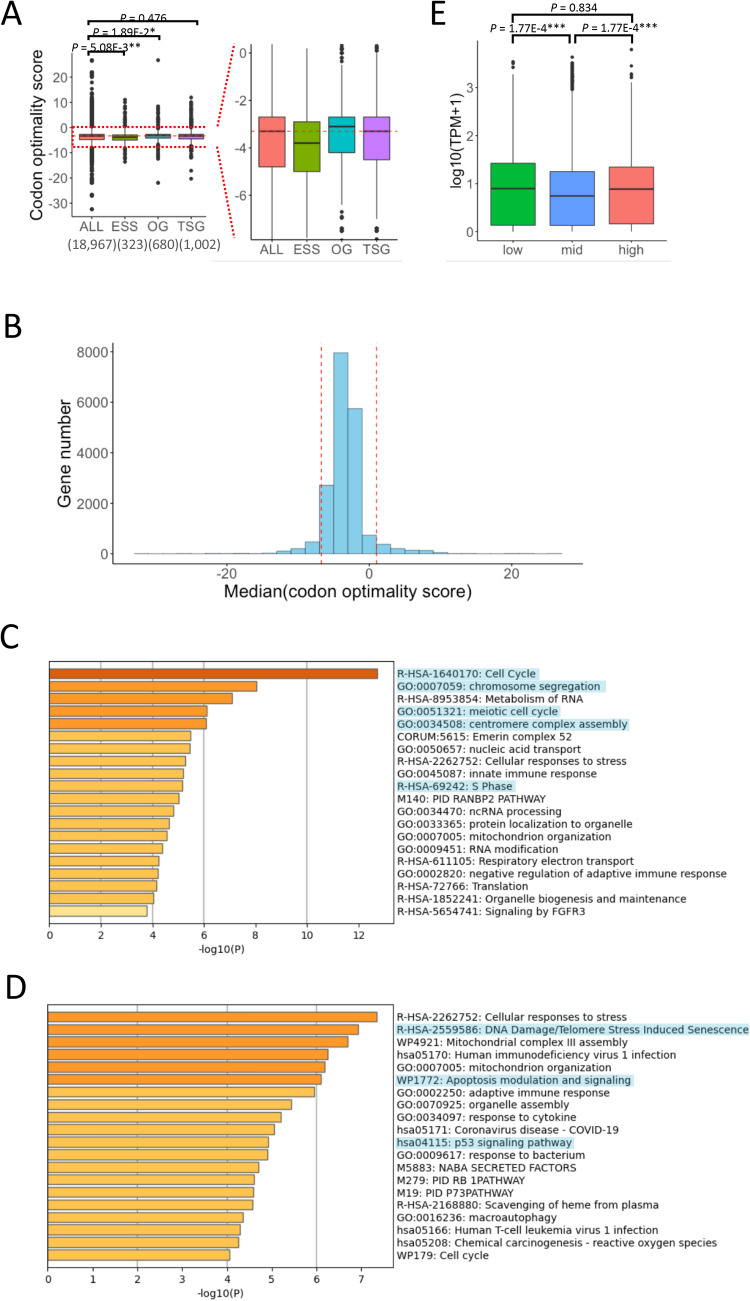
Characteristics of genes with silent mutations. (**A**) The codon optimality scores of silent mutations in various genes. The horizontal axis represents four kinds of gene lists (ALL: all genes, ESS: essential genes, OG: oncogenes, and TSG: tumor suppressor genes). (**B**) The distribution of mean values per gene of codon optimality scores for silent mutations. The horizontal axis represents the mean values per gene. The vertical axis represents the number of genes. The red dotted lines represent the top and bottom 5% of the mean values. (**C**) Gene Ontology (GO) enrichment analysis of the top 5% genes. Highlighted GO terms represent terms related to cell cycle and cell division. (**D**) GO enrichment analysis of the bottom 5% genes. Highlighted GO terms represent terms related to apoptosis and cellular senescence. (**E**) The expression levels of genes with silent mutations. The horizontal axis represents three groups based on codon optimality scores of silent mutations (low: bottom 5%, high: top 5%, and mid: the other genes).

Silent mutations that give rise to high-frequency codons are enriched in oncogenes, while those that lead to low-frequency codons are enriched in tumor suppressor genes (TSGs)^9–11^. To confirm this result, we used three kinds of gene lists—essential genes comprising housekeeping genes (331), oncogenes (803), and TSGs (1217)—and calculated codon optimality scores for the silent mutations. We found that compared with whole genes, silent mutations in the essential genes significantly decreased their codon optimality scores, those in the oncogenes significantly increased their scores, and those in the TSGs showed no significant differences (Fig. 5A). This may be derived from the different cancer-related gene data and the different strategy employed in this study to compare codon optimality of silent mutations^9–11^.

Then, we focused on silent mutations with extremely high or low codon optimality scores and investigated whether the function of the corresponding genes was associated with cancer pathogenesis. First, we calculated the median codon optimality scores of the mutations in each gene. The genes in the top and bottom 5% of the median codon optimality scores were extracted (Fig. 5B). The top 5% (median codon optimality score ≥ 1.00) contained 959 genes, while the bottom 5% (median codon optimality score ≤ − 6.85) contained 945 genes. Gene ontology (GO) analysis using Metascape was performed on these genes^32^. We found that genes related to the cell cycle and cell division were enriched among the top 5%, whereas genes related to apoptosis and cell senescence were enriched among the bottom 5% (Fig. 5C,D). Namely, optimal silent mutations are more likely to occur in genes associated with cancer development, while non-optimal silent mutations are more likely to occur in genes associated with cancer suppression. These results support the hypothesis that part of silent mutations can cause changes in translation processes, such as mRNA stability, translation efficiency, and protein folding, leading to the growth and proliferation of cancer cells.

If we assume that silent mutations truly influence translation processes, genes that are likely to be affected by them should be those with higher expression levels. To evaluate this hypothesis, first, we compared the gene expression levels among the three groups segregated based on their median codon optimality scores in the GO analysis (low: 945 genes, high: 959 genes, mid: other genes). We found that genes in the high and low groups were expressed at a significantly higher level than those in the mid group (Fig. 5E). Next, we further divided the high and low groups into two subgroups each, based on whether the median gene expression levels were log_10_(TPM) ≥ 1 or < 1 (Supplementary Fig.  S4A). We found that genes related to the cell cycle and cell division were more strongly enriched in the higher expression group with optimal mutations, whereas genes related to apoptosis were more strongly enriched in the higher expression group with non-optimal mutations (Supplementary Fig. S4B–E).

To evaluate whether the genes affected by silent mutations change during cancer evolution, we subdivided the high and low groups (described above) into two each, based on whether the mutation was early or late. We found that genes related to the cell cycle and cell division predominantly harbored early optimal mutations, whereas those related to apoptosis and suppression of cell proliferation mainly carried early non-optimal mutations (Supplementary Fig. S5A–D). Taken together, part of silent mutations with extremely high or low codon optimality scores in highly expressed genes that occurred early during cancer evolution can potentially affect carcinogenesis, cancer growth, and proliferation.

## Discussion

In this study, we used WES data on 33 cancer types obtained from TCGA to comprehensively analyze silent mutations, which have been less studied than missense mutations. Usually regarded as neutral or nearly neutral and less important in the context of cancer^1^, our study showed that some silent mutations can potentially be harmful and that the number of optimal silent mutations (those that give rise to high-frequency codons) is likely to increase as cancer evolves. The position of optimal and non-optimal silent mutations (those that give rise to low-frequency codons) depends on the surrounding codons: they are likely to occur in regions abundant in low- and high-frequency codons, respectively. Optimal silent mutations are more likely to occur in genes involved in cancer growth and proliferation, while non-optimal silent mutations are more likely to occur in cancer-suppressing genes. These results suggest that silent mutations, like missense mutations, may directly be involved in cancer pathogenesis. They can influence mRNA stability, translation efficiency, and protein folding of cancer-related genes and proteins^3^, supporting cancer growth, invasion, and metastasis. However, the impact of the silent mutations identified in our study on cancer might not be substantial, and they may potentially contribute to cancer progression in concert with other accumulated mutations.

**Figure 6:**
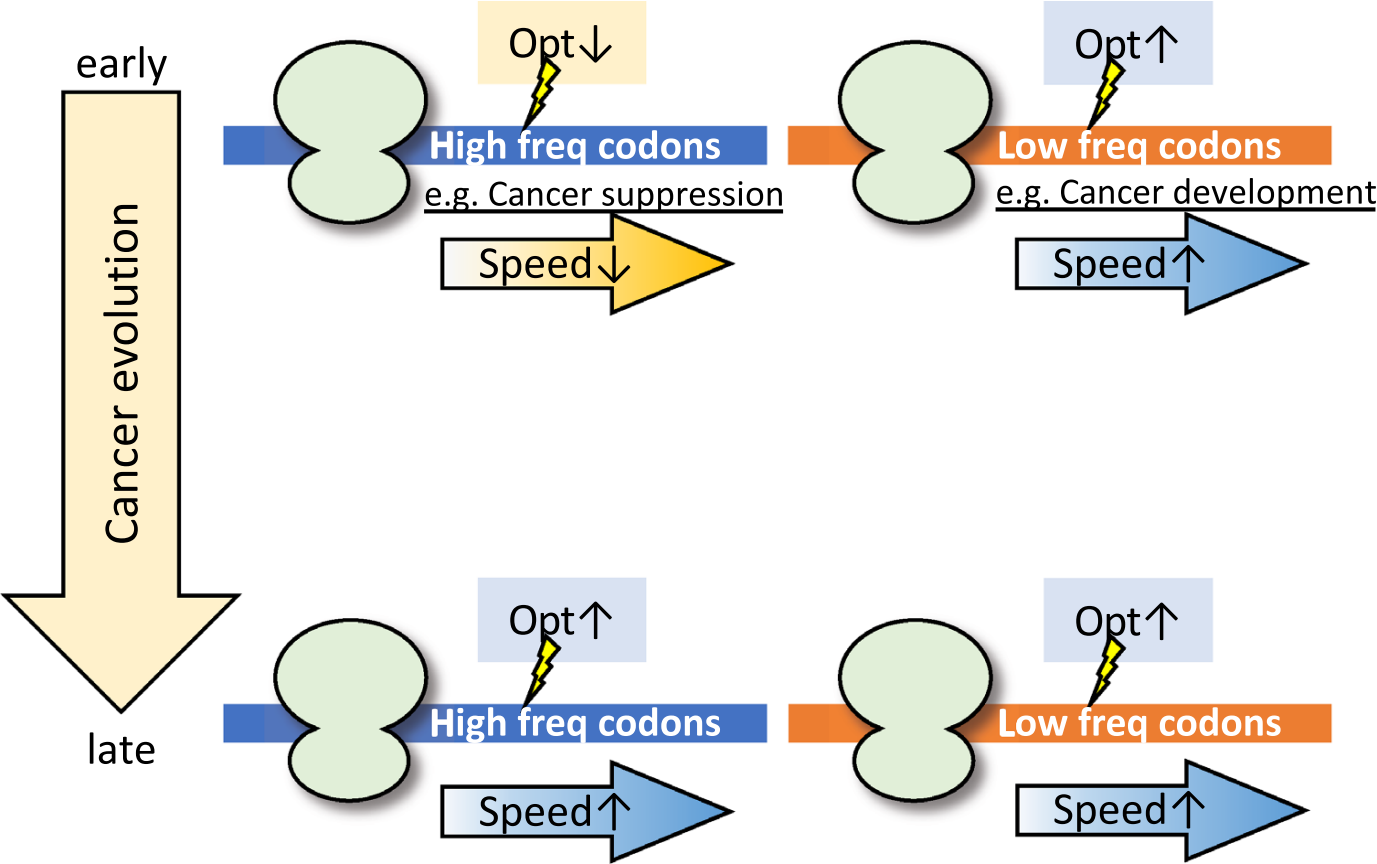
Silent mutation model in cancer evolution. The model of silent mutation in cancer evolution based on this study. The figure depicts a ribosome translating mRNA. The blue and orange horizontal lines indicate mRNAs in regions with high and low codon frequencies, respectively. “Opt↑” represents silent mutations with a high codon optimality score (optimal mutation), and “Opt↓” represents silent mutations with a low codon optimality score (non-optimal mutation). “Speed↑” represents increased translation efficiency, and “Speed↓” represents decreased translation efficiency.

Based on our results, we propose the silent mutation model in cancer evolution (Fig. 6). In the early stages of tumorigenesis, silent mutations that decrease codon optimality are likely to occur in sequences rich in high-frequency codons, while those that increase codon optimality are likely to occur in sequences rich in low-frequency codons. Some of these genes are involved in cancer suppression and cancer development, respectively. Therefore, silent mutations may potentially promote cancer growth and proliferation by modulating the translation of these genes. In the late stage of tumorigenesis, silent mutations that increase codon optimality are likely to occur, regardless of the surrounding sequences. They are not enriched in genes with specific functions. Therefore, these mutations likely help meet the high translational demands of the cancer cell. These results suggest that silent mutations are not always neutral; some of them may support the drastic growth and proliferation of cancer cells.

For the analysis of codon usage bias in this study, we used the codon usage table, which is the most suitable metric to compare among amino acids and among cancer types. There are several other metrics than the codon usage table. The codon adaptation index^33^ is a simple and widespread metric of the overall synonymous codon usage bias of a gene. For the calculation of CAI in each gene, we use the geometric mean of the relative adaptiveness of all the codons in the gene. Because the adaptiveness is a relative value among identical amino acids, it is not suitable for comparing the values among different amino acids. The tRNA adaptation index^34^ is a metric of translational efficiency for a gene. It is calculated using the abundance of intracellular tRNA and the binding strength between a codon and a tRNA. Because the tRNA exhibits tissue-specific expression^35^, tAI is not suitable for the analysis of mutation data derived from various tissues. The codon stabilization coefficient^4^ indicates mRNA stability and is derived from the correlation between each codon frequency in transcripts and mRNA half-life experimental data. Because cancer cell-derived mRNA half-life data is not available, CSC is not suitable for cancer research. Ribosome profiling^36^ can provide information on the efficiency of ribosomal translation. Currently available public ribosome profiling data are mainly derived from cancer cell lines that do not cover the tissues of all cancer types used in this study. We need to carefully consider these various metrics and perform an integrated analysis for codon usage bias in the future.

This study has some limitations. We could not directly evaluate the effects of silent mutations on translation levels because proteome data, WES data, and RNA-Seq data with a sufficient sample size from identical samples were not available. We were also unable to investigate the effects of silent mutations on translation efficiency or speed because ribosome profiling data derived from the samples in TCGA was unavailable. Hence, we could only confirm the effects on gene expression levels. We believe that the accumulation of translation-related data in the future will enable us to comprehensively verify the relationship between silent mutations and translation levels of the corresponding genes in cancer cells.

## Conclusions

Although cancer genome analysis has mainly focused on mutations and sometimes on transcripts, evaluating the effects of mutations on translation processes and products is equally important. This study sheds light on the salient effects of silent mutations on translation processes in cancer cells. A comprehensive analysis of changes accompanying each step of DNA to mRNA to protein in the context of mutations in a cancer cell would provide unprecedented insights into cancer pathogenesis.

## Methods

### Datasets

WES data were downloaded from TCGA^37^. The data was obtained through the MC3 Project^15^ and were derived from 33 cancer types (Supplementary Table 1). Gene expression quantification data, calculated as TPM, and copy number variant data, annotated by the ASCAT2 pipeline^38^, were downloaded from The Centers for Disease Control and Prevention portal in the National Cancer Institute. Purity and ploidy data were downloaded from https://gdc.cancer.gov/about-data/publications/pancanatlas. SNP data were downloaded from gnomAD (version 2.1.1)^39^. Gene annotation data for human (Homo_sapiens.GRCh37.87.gtf) was downloaded from Ensembl. In this study, the human reference genome GRCh37 was used.

### Calculation of codon optimality score for silent mutations

Each codon carrying a silent mutation, as annotated by the MC3 Project^15^, was assigned a codon frequency (per 1000 triplets) using the *Homo sapiens* codon usage table available from the Kazusa Research Institute^40^. The frequency of a codon with mutation (alt) minus its frequency without mutation (ref) was defined as the codon optimality score in this study. For SNPs, we extracted codons with silent mutations using SNPeff (5.1d).

### Evolutionary conservation of silent mutations

PhastCons (primates.phastCons46way.bw) and PhyloP score data (primates.phyloP46way.bw) were downloaded from UCSC Genome Browser. These data are derived from multiple alignments of 10 primates (human, chimp, gorilla, orangutan, rhesus, baboon, marmoset, tarsier, mouse, lemur, and bushbaby). We extracted PhastCons scores and PhyloP scores of silent mutations from these datasets to compare their evolutionary conservation. We used 100,000 bases randomly selected from coding CDSs as a control for the comparison.

### Evolutionary history of silent mutations

The relative timing of silent mutations to copy number gains was estimated using R package MutationTimeR (v.1.0.2)^17^. For the calculation, we used purity data, copy number variant data, and variant call format files created from somatic mutations of each corresponding sample. MutationTimeR classifies mutations into one of four groups: early clonal, late clonal, clonal, and subclonal. We defined early and clonal mutations as “early mutations” and late clonal and subclonal mutations as “late mutations.”

### Frequencies of the codons upstream and downstream of a silent mutation

Protein CDS data annotated by the CCDS project^41^ was downloaded from CCDS Database. From this data, we extracted 30 bases (10 codons) each, upstream and downstream of the codons with silent mutations, and assigned them codon frequencies as described above. We calculated the average frequencies in the 10 codons upstream, in the 10 codons downstream, and in both of them together.

### Functional analysis of genes with silent mutations

We used three different gene lists to analyze the association between gene function and codon optimality score: 331 essential genes^42^ that were defined based on their housekeeping function and evolutionary conservation, 803 oncogenes registered in the oncogene database (http://ongene.bioinfo-minzhao.org/download.html), and 1217 TSGs registered in TSGene 2.0^43^. GO analyses were performed using Metascape (v.3.5)^32^. To focus on genes in which silent mutations are prone to be accumulated, only those with ≥ 5 mutations were selected.

### Statistical analyses

Statistical analyses were performed using the R software version 4.0.1 (R Project for Statistical Computing, Vienna, Austria). Wilcox rank sum test was used to compare values between two groups. Benjamini–Hochberg procedure was used to adjust *P*-values for multiple testing^44^. Two-tailed tests of significance were used. A *P*-value of < 0.05 was considered statistically significant (*P < 0.05, **P < 0.01, and ***P < 0.001).

## Supplementary Information

Supplementary Figures.

Supplementary Table 1.

Supplementary Table 2.

## Data Availability

All datasets are freely available from public databases. The results shown here are mainly based on data generated by TCGA: https://portal.gdc.cancer.gov/. We also used SNP data from gnomAD: https://gnomad.broadinstitute.org/, Protein CDS data from CCDS Database: https://www.ncbi.nlm.nih.gov/projects/CCDS/CcdsBrowse.cgi, codon usage table from the Kazusa Research Institute: https://www.kazusa.or.jp/codon/cgi-bin/showcodon.cgi?species=9606&aa=1&style=N and UCSC genome browser: http://genome.ucsc.edu/. The datasets and scripts generated in the present study are available from the corresponding author upon reasonable request. Further information and requests for codes and scripts generated in the present study should be directed to and will be fulfilled by the Lead Contact, Mikita Suyama (mikita@bioreg.kyushu-u.ac.jp).

## Abbreviations

CCDS project: Consensus CDS project
CDS: Coding sequence
GO: Gene ontology
MC3 project: Multi-Center Mutation Calling in Multiple Cancers project
SNP: Single nucleotide polymorphism
TCGA: The Cancer Genome Atlas
TPM: Transcripts per kilobase million
TSG: Tumor suppressor gene
WES: Whole exome sequencing
