# Supplementary material for: Possible involvement of silent mutations in cancer pathogenesis and evolution
Source: Sci Rep. 2023 May 10;13:7593. doi: 10.1038/s41598-023-34452-w (PMC10172315; doi:10.1038/s41598-023-34452-w)
Supplement: Supplementary file 1 — Supplementary Figures. [file 41598_2023_34452_MOESM1_ESM.pdf]

Fig. S1
C Kikutake

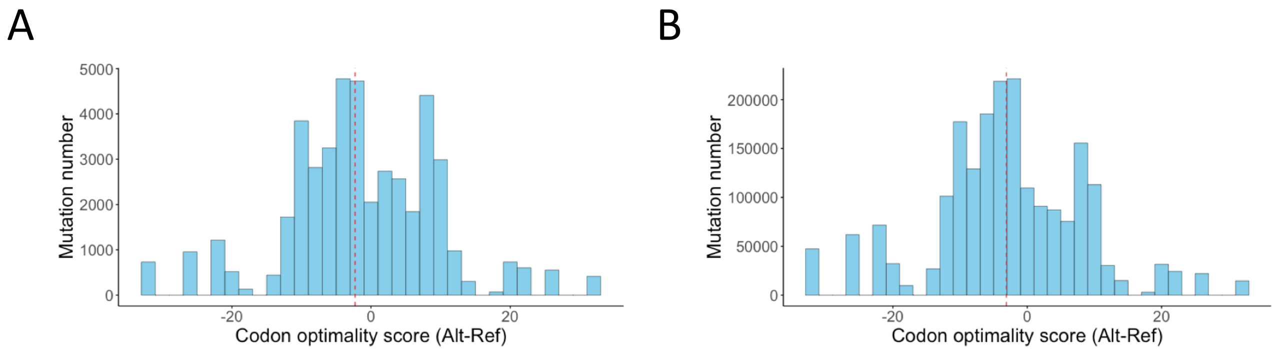

Supplementary Figure S1. Distribution of codon optimality scores for each silent single nucleotide polymorphism (SNP).

(A) The distribution of codon optimality scores of each silent SNP (allele frequency ≥ 0.01).
(B) The distribution of codon optimality scores of each silent SNP (allele frequency <0.01).

Fig. S2
C Kikutake

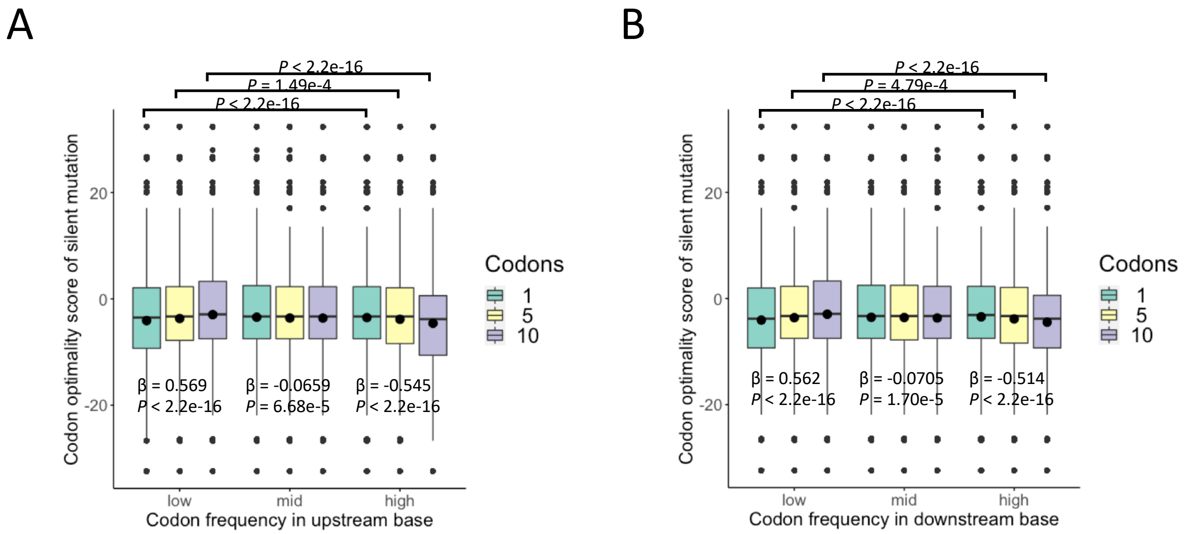

Supplementary Figure S2. Upstream and downstream nucleotide context of silent mutations.

(A) The mean codon frequencies (per 1,000 triplets) of 1, 5, and 10 codons upstream of silent mutation-carrying codons and their codon optimality scores. The horizontal axis represents three groups based on the mean codon frequencies of the 1, 5, and 10 codons (low: bottom 25%, mid: 25%–75%, and high: top 25%). β represents the partial regression coefficient by multiple regression analysis using the codon optimality scores in each of the three groups. (B) The mean codon frequencies of 1, 5, and 10 codons downstream of silent mutation-carrying codons and their codon optimality scores.

Fig. S3
C Kikutake

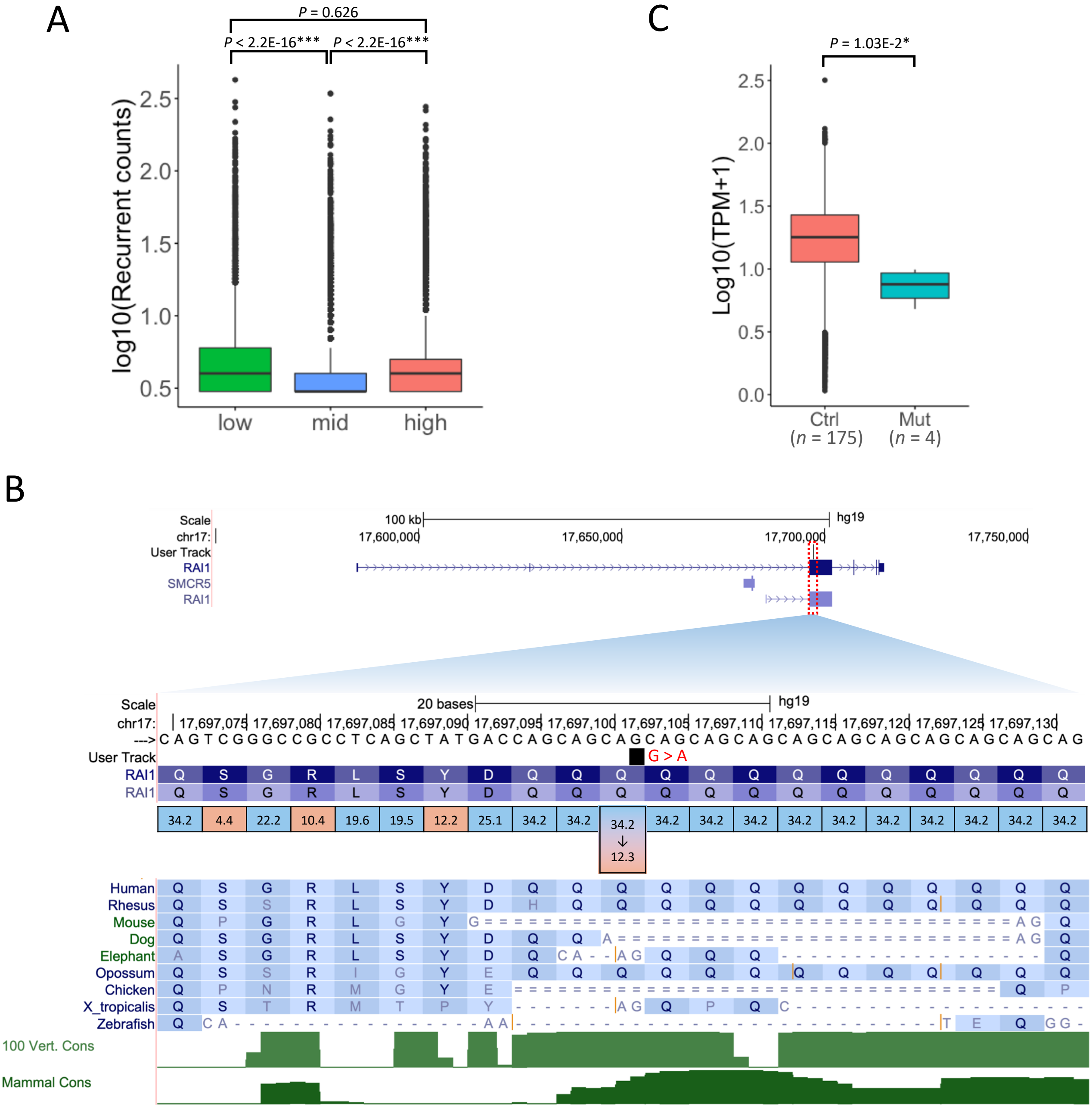

Supplementary Figure S3. Extraction of silent mutation associated with cancer pathogenesis.

(A) The position of the extracted silent mutation in RAI1. The figure shows the nucleotide sequence, amino acid sequence, codon optimality, and evolutionary conservation status. (B) The expression levels of RAI1 with and without silent mutations. The figure shows the nucleotide sequence, amino acid sequence, codon optimality, and evolutionary conservation status. Numbers in squares are codon frequencies (per 1,000 triplets). The blue squares indicate codons ≥15.1 (median frequency) and the red squares indicate codons <15.1. (C) The recurrent counts of silent mutations per gene. The horizontal axis represents three groups based on codon optimality scores of the silent mutations (low: bottom 5%, high: top 5%, and mid: the other genes).

Fig. S4
C Kikutake

A

Optimality / Expression	High average optimality score	Low average optimality score
High expression $\log_{10}(TPM) \geq 1$	355 genes	387 genes
Low expression $\log_{10}(TPM) < 1$	507 genes	475 genes

B

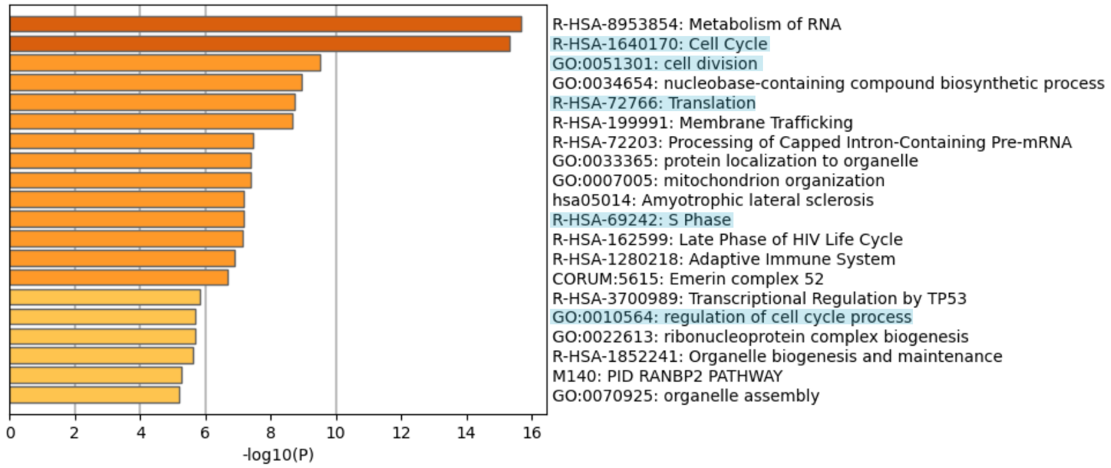

C

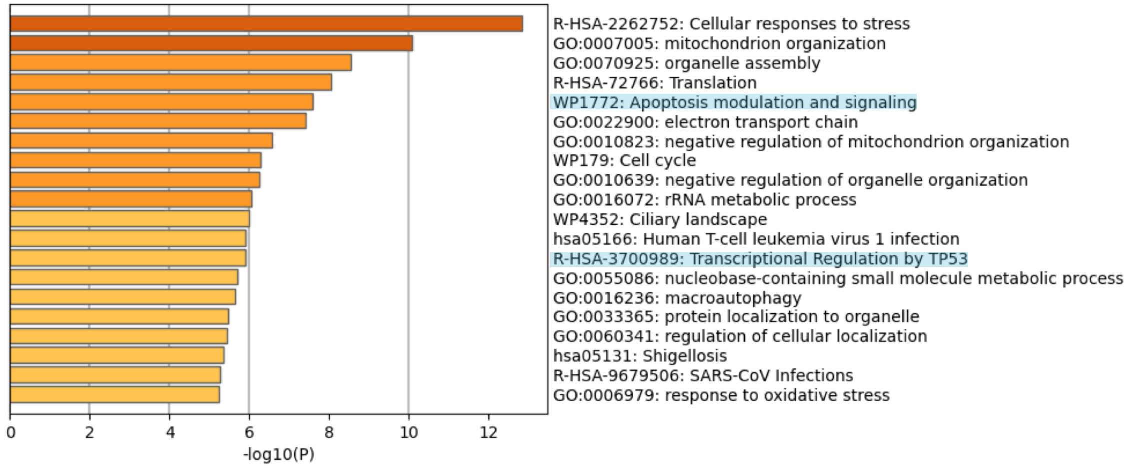

Fig. S4
C Kikutake

D

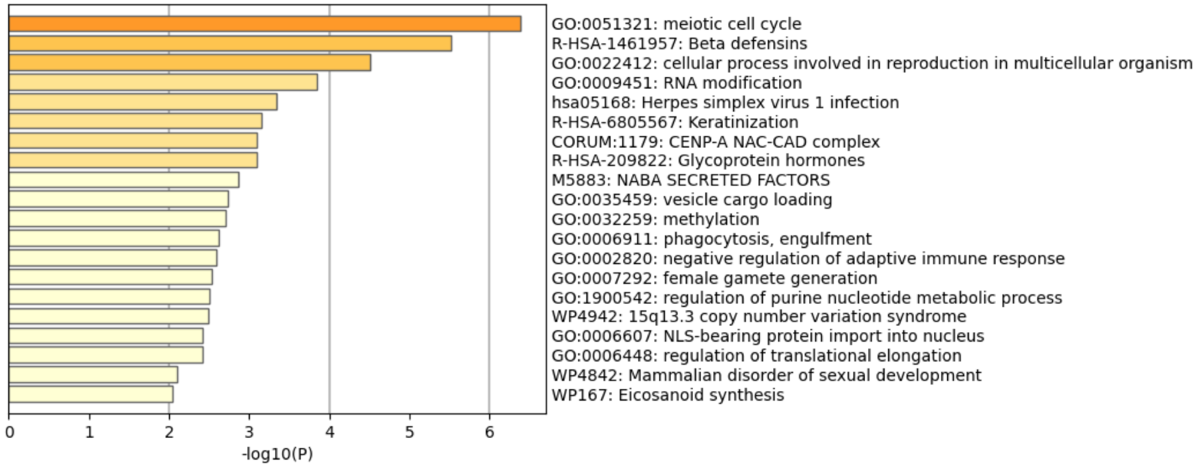

E

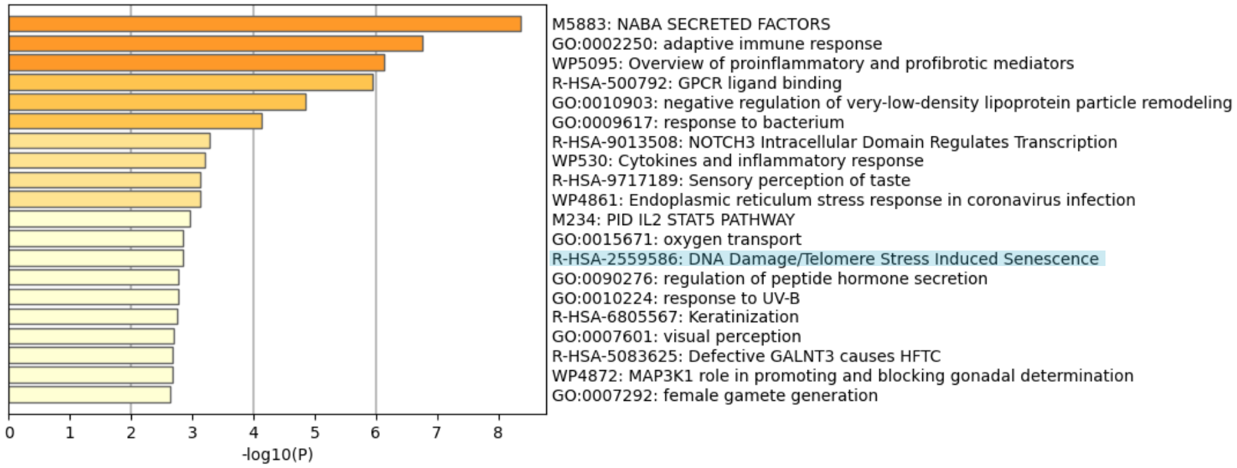

Supplementary Figure S4. Characteristics of high- and low-expressed genes with silent mutations.

(A) The number of genes in each of the four groups based on the mean codon optimality scores and gene expression levels. (B) GO enrichment analysis of the top 5% genes and those with higher expression levels log10(transcripts per kilobase million [TPM]) ≥ 1. Highlighted GO terms represent terms related to the cell cycle and cell division. (C) GO enrichment analysis of the bottom 5% genes with bottom 5% and with those with higher expression levels log10(TPM) ≥ 1. Highlighted GO terms represent terms related to apoptosis. (D) GO enrichment analysis of the top 5% genes and those with lower expression levels log10(TPM) <1. Highlighted GO terms represent terms related to the cell cycle and cell division. (E) GO enrichment analysis of the bottom 5% genes and those with lower expression levels log10(TPM) <1. Highlighted GO terms represent terms related to cellular senescence.

Fig. S5
C Kikutake

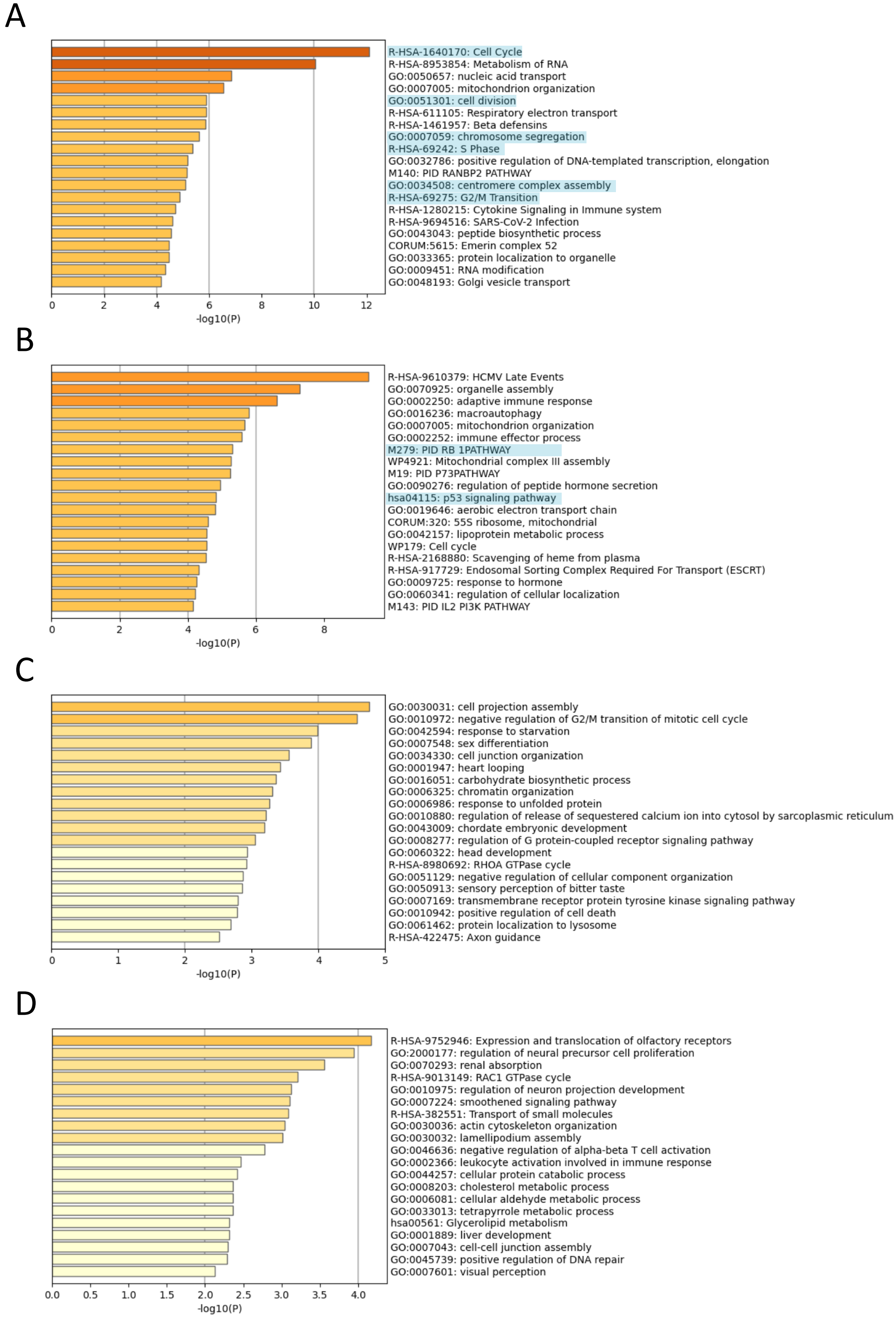

Fig. S5
C Kikutake

Supplementary Figure S5. Characteristics of genes with early clonal and subclonal mutations.

(A) GO enrichment analysis of early clonal (classified as clonal [early]) top 5% genes. Highlighted GO terms represent terms related to the cell cycle and cell division. (B) GO enrichment analysis of early clonal (classified as clonal [early]) bottom 5% genes. Highlighted GO terms represent terms related to apoptosis and suppression of cell proliferation. (C) GO enrichment analysis of subclonal (classified as subclonal) top 5% genes. (D) GO enrichment analysis of subclonal (classified as subclonal) bottom 5% genes.
